# Caveolin-1 Dependent Endocytosis Enhances the Chemosensitivity of HER-2 Positive Breast Cancer Cells to Trastuzumab Emtansine (T-DM1)

**DOI:** 10.1371/journal.pone.0133072

**Published:** 2015-07-14

**Authors:** Yuan-Chiang Chung, Jang-Fang Kuo, Wan-Chen Wei, King-Jen Chang, Wei-Ting Chao

**Affiliations:** 1 Department of Surgery, Cheng-Ching General Hospital, Chungkang Branch, Taichung, Taiwan; 2 Department of Pathology, Cheng-Ching General Hospital, Chungkang Branch, Taichung, Taiwan; 3 Department of Life Science, Tunghai University, Taichung, Taiwan; University of South Alabama, UNITED STATES

## Abstract

The humanized monoclonal antibody-drug conjugate trastuzumab emtansine (T-DM1, Kadcyla) has been approved by the U.S. FDA to treat human epidermal growth factor receptor 2 (HER-2)-positive metastatic breast cancer. Despite its effectiveness in most patients, some are initially resistant or develop resistance. No biomarker of drug resistance to T-DM1 has been identified. Antibody-drug efficacy is associated with antibody internalization in the cell; therefore, cellular sensitivity of cells to the drug may be linked to cellular vesicle trafficking systems. Caveolin-1 is a 22 KD protein required for caveolae formation and endocytic membrane transport. In this study, the relationship between caveolin-1 expression and the chemosensitivity of HER-2-positive breast cancer cells to T-DM1 was investigated. Samples from 32 human breast cancer biopsy and normal tissue specimens were evaluated immunohistochemically for caveolin-1 expression. Caveolin-1 was shown to be expressed in 68% (22/32) of the breast cancer specimens. In addition, eight (72.7%, 8/11) HER-2 positive breast cancer specimens had a higher caveolin-1 expression than normal tissues. HER-2-positive BT-474 and SKBR-3 breast cancer cells that express low and moderate levels of caveolin-1, respectively, were treated with trastuzumab or its conjugate T-DM1. Cell viability and molecular localizations of caveolin-1, antibody and its conjugate were examined. Confocal microscopy showed that T-DM1 and caveolin-1 colocalized in SKBR-3 cells, which also were five times more sensitive to the conjugate in terms of cell survival than BT-474 cells, although T-DM1 also showed improved drug efficacy in BT-474 cells than trastuzumab treatment. Caveolin-1 expression in these lines was manipulated by transfection of GFP-tagged caveolin-1 or caveolin-1 siRNA. BT-474 cells overexpressing caveolin-1 were more sensitive to T-DM1 treatment than mock-transfected cells, whereas the siRNA-transfected SKBR-3 cells had decreased sensitivity to T-DM1 than mock-transfected SKBR-3 cells. The expression of caveolin-1 could mediate endocytosis and promote the internalization of T-DM1 into HER-2 positive cancer cells. Thus, caveolin-1 protein may be an effective predictor for determining the outcome of T-DM1 treatment in breast cancer patients.

## Introduction

Human epidermal growth factor receptor 2 (HER-2) has been identified as oncoprotein in breast cancer. The overexpression of HER-2 mRNA and protein occurs in 20–30% of invasive breast cancers and is a predictor of poor clinical outcome [1, 2]. The humanized monoclonal antibody, trastuzumab (Herceptin), binds to the extramembrane domain of HER-2 to inhibit the proliferation and survival of HER-2 dependent tumors. After several effective trials, in 2001, trastuzumab was approved by the Food and Drug Administration (FDA) in the USA for patients with advanced breast cancers that express HER-2 [3]. Despite the success of this therapeutic treatment, naked trastuzumab targeting of HER-2 expression in breast cancer is rarely curative by itself, and most of the effects of this drug are achieved in combination with chemotherapy [4–7]. However, there are adverse effects of combination therapy: 27% of patients treated concurrently with trastuzumab and anthracyclines, and 13% with trastuzumab and paclitaxed, had cardiotoxic side effects [8].

Recent advances in antibody drug conjugate (ADC) techniques allow for the linkage of specific monoclonal antibodies with potent cytotoxic drugs to reduce systemic toxicity and increase therapeutic benefits in cancer patients [9, 10]. HER-2-based ADC targeting have been investigated for clinical application in breast cancer treatment [11, 12] using trastuzumab emtasime (trastuzumab-DM1; T-DM1), in which trastuzumab is conjugated through a stable thioether bond to the maytansanoid derivative emtasine. The latter has potent anti-mitotic effects by preventing microtubule assembly. The antibody portion of the conjugate binds to the HER-2 receptor on the surface of cancer cells allowing for the internalization of T-DM1 and its subsequent degradation to release the lethal drug [11]. Under this mechanism, T-DM1 treatment allows patients a better quality of life by preventing the toxic effects of microtubule-targeting chemotherapy. In the phase III of the EMILIA trail, T-DM1 significantly prolonged progression-free and overall survival with less toxicity than the dual tyrosine kinase inhibitor lapatinib plus capecitabine in patients with HER-2 positive advanced breast cancer [13]. The National Comprehensive Cancer Network Guidelines (NCCN) approved T-DM1 application in metastatic HER-2 positive breast cancer in 2013. Despite the efficacy of T-DM1, most patients treated with this ADC eventually progress to more serious stages of disease [13–15], and some HER-2 positive breast cancers are primarily non-responsive or only have a minimal response to T-DM1. In the EMILIA trial conducted in a second-line setting with a patient population who had previously been treated with trastuzumab and taxane, 228 (66%) of the 397 patients treated with T-DM1 did not have an objective response [13]. In addition, in the TDM4450g trial carried out in a first line setting, 46% of metastatic breast cancer patients did not respond to T-DM1 treatment [14]. Factors that are strongly implicated in the biological mechanisms of T-DM1 action are likely to play a role in resistance to T-DM1. One such factor is the triggering entry of the HER-2-T-DM1 conjugate into cancer cells via receptor-mediated endocytosis [9, 10, 16, 17]. High efficiency of conjugate internalization may result in a high intracellular concentration of DM1, whereas deceleration of endocytosis might cause a loss of sensitivity to T-DM1. Therefore, understanding the mechanisms of antibody internalization could lead to the discovery of a biomarker for ADC treatment outcome; especially with regard to T-DM1 treatment.

Internalization of antibody with targeted growth factor receptor through vesicle trafficking systems has been proposed to be associated with drug sensitivity [9, 17]. Cellular vesicle traffic systems function in protein transport, membrane receptor recycling, and protein degradation. Vesicle transport mediated through the endocytic system, including endocytosis and recycling, is controlled primarily by membrane endocytotic vesicles and small GTPases of the Rab family [18]. Clathrin is recognized as a membrane endocytic protein for the transport of molecules from the membrane into the cell [19]. Receptor tyrosine kinases (RTKs) such as epidermal growth factor receptor (EGFR) have been shown to be involved in internalization through the clathrin-dependent endocytosis pathway; however, a clathrin-independent mechanism, such as caveolin-1 for RTKs internalization in breast cancer, has also been demonstrated [20, 21]. Caveolin-1 is the 22 KD membrane protein that is required for caveolae structure formation and intracellular caveosome transport [22, 23]. The vesicle traffic route for membrane receptors may vary depending on cell types and genetic variation. Previous reports have suggested a negative regulatory role for caveolin-1 in tumor development in a number of human cancers [24, 25], and recent studies proved that caveolin-1 inhibits breast cancer cell migration and metastasis [26–29]. Trastuzumab is internalized via endocytosis, but more than 50% is recycled back to the membrane [30]. In a recent report, trastuzumab was demonstrated to colocalize with caveolin-1 in breast cancer cells [31]. However, whether T-DM1 uses the same internalization mechanism as trastuzumab is still unknown. Poor internalization has been considered an important factor leading to primary or acquired resistance in T-DM1 treatment of metastatic breast cancer [32].

The particular trafficking route used affects the half-life of an ADC inside the cell and further influences the drug efficacy. Therefore, deciphering the mechanism of T-DM1 internalization is critical for its clinical application in advanced precision therapy. This study investigated the mechanism of T-DM1 internalization and the effect of this mechanism on drug efficacy. The results will help to identify vesicle trafficking markers for clinical T-DM1 application.

## Materials and Methods

### Patients and ethics statements

The study group consisted of 32 female patients (age range 35–85 years old, median age 48.5 years old) who had undergone resection for localized breast cancer between April 2011 and December 2013 at Cheng-Ching General Hospital, Taiwan. The protocol was reviewed and approved by the Cheng-Ching General Hospital Institutional Review Board (HT110018). Written informed consent was obtained from all patients. Three patients were in stage I, 13 patients were in stage II, and 16 patients were in stage III of breast cancer. Tumor histology grade, specific hormone and HER-2 receptor expressions, tumor size, tumor presence or absence in lymph nodes, and pathological stage were recorded. All assessments in this study were carried out without knowledge of pathological or surgical data.

### Immunohistochemistry

**Fig 1:**
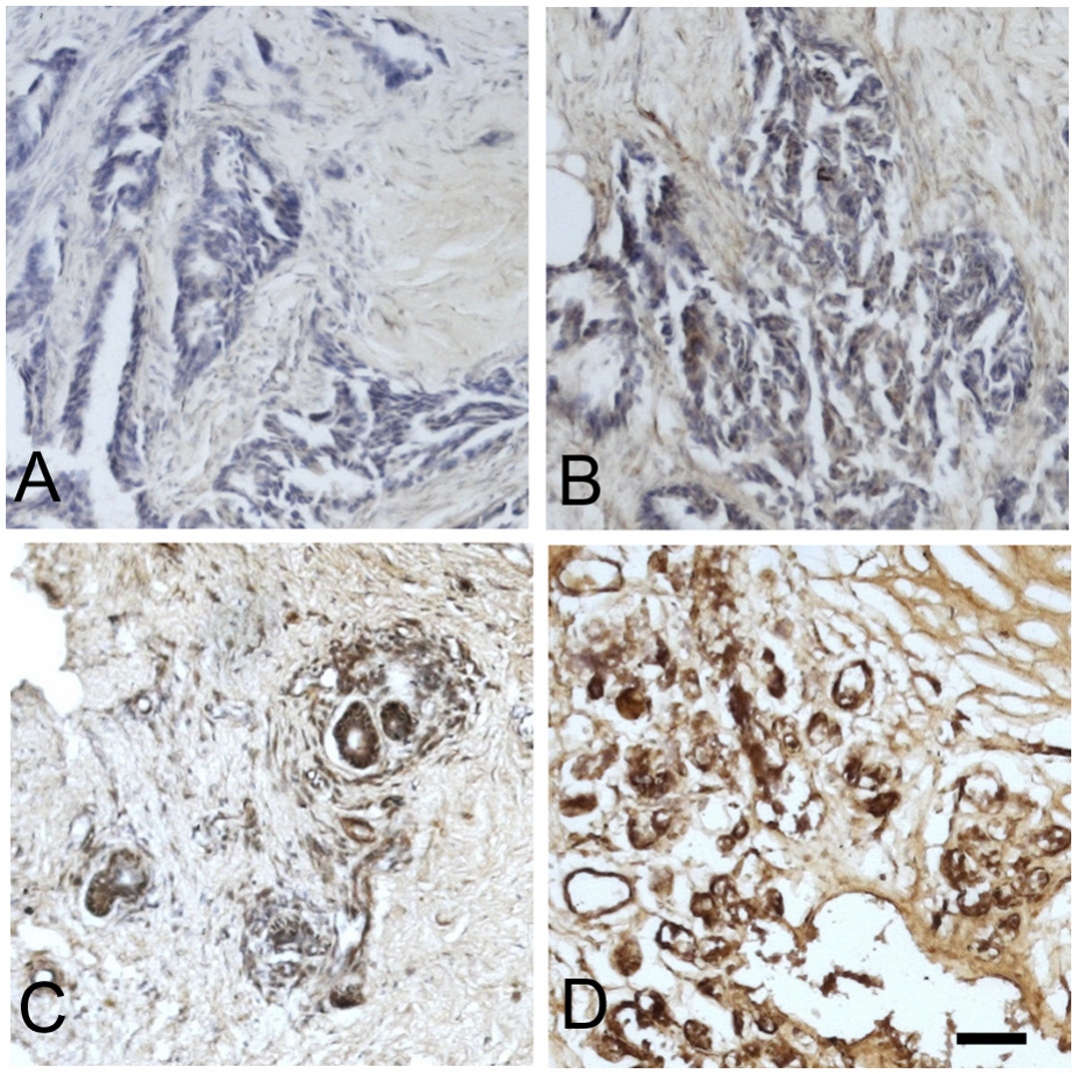
Immunohistochemical staining of caveolin-1 in breast cancer tissues. (A) Negative staining; (B) weak staining; (C) moderate staining; (D) heavy staining. Scale bar = 100 μm.

The specimens with breast epithelial tissue (tumor site and non-tumor site) of patients with breast cancer were proceeded for immunohistochemistry. Five-μm-thick paraffin sections were incubated with anti-caveolin-1 (1:100) or anti-HER-2 (1:1000) antibodies (Dako, Carpinteria, CA, USA) at room temperature for 1 hr. A histostain-SP diaminobenzidine tetrahydrochloride (DAB) kit (Invitrogen, ThermoFisher, Waltham, MA, USA) was then used to visualize the primary antibody. For HER-2, the strength of the membranous staining was graded on 4-point scale from 0 to 3 and only 3+ was considered positive according to HercepTest guideline from Dako. The scoring used for caveolin-1 by immunohistochemistry was the “I” index [33]; the equation for which is I = 0×f0 + 1×f1 + 2×f2 + 3×f3, where f0-f3 are the fractions of the cells showing a defined level of staining intensity (from 0–3). The numbers 0–3 represent the following: "0" negative, no detectable staining, "1" weak, but still detectable staining, "2" moderate, clearly positive but still weak; and "3" heavy and intense staining (Fig 1). Scores ranged from 0 to 3. A score greater than the mean value of 1.5 was considered as high expression, and a score less than 1.5, as low expression of caveolin-1 [34, 35].

### Cell lines and transfection

To investigate the internalization of HER-2-based antibodies, cultured breast cancer cells with or without HER-2 expression were used. In this study, the triple negative breast cancer MDA-MB-231 cell line, and MCF-7 cell line were used as HER-2 negative cells, and SKBR-3, BT-474, and ZR-75-1 were used as HER2 positive cells, whereas ER (estrogen receptor) and PR (progesterone receptor) expression by Allred scores were (0, 0) in MDA-MB-231, (6, 6) in MCF-7, (0, 0) in SKBR-3, (0, 8) in BT-474 and (3, 4) in ZR-75-1 cells [36]. Cell lines were provided as gift by Dr. Wang LH in Institute of Molecular and Genomic Medicine of National Health Research Institutes, Miaoli, Taiwan. Cells were grown in Dulbecco's modified Eagle's Medium (DMEM) supplemented with 10% calf serum, penicillin, and streptomycin (GIBCO-BRL, Gaithersberg, MD, USA) and were kept in an incubator with 5% CO_2_ at 37°C. For transfection, BT-474 cells were grown on 24-well plates in normal growth medium without antibiotics, and Lipofectamine 2000 transfection reagent (Invitrogen, ThermoFisher, Waltham, MA, USA) was used to induce GFP-tagged caveolin-1 (Addgene, Cambridge, MA, USA) overexpression. Caveolin-1siRNA (Dharmacon, GE health care, Lafayette, CO, USA) was used in the knockdown experiment in SKBR-3 cells. Cells were analyzed 24 hr post-transfection, and the efficacy of transfection was confirmed by immunoblot analysis of cell lysates using rabbit anti-GFP (abcam, Cambridge, MA, USA) and rabbit anti-caveolin-1 (Santa Cruz, Dallas, TX, USA) antibodies.

### Western blot

Cells were scraped in lysis buffer (1% NP-40, 50 mM Tris pH 7.4, 150 mM NaCl, 2 mM MgCl_2_, 1 mM EGTA, and protease and phosphatase inhibitors) on an ice tray, and the protein concentration was determined by bicinchoninic acid (BCA) assay. Protein samples were mixed with sample buffer, boiled for 5 min and separated by SDS—PAGE. Proteins on the gel were then transferred onto a PVDF membrane, blocked in blocking buffer containing 5% bovine serum albumin (BSA), and then probed with primary antibodies against caveolin-1, clathrin, Bcl-2, and GAPDH (Santa Cruz, Dallas, TX, USA), followed by incubation with appropriate HRP-conjugated secondary antibodies. Blots were developed using an enhanced chemiluminescence system.

### Confocal microscopy for the detection of antibody internalization

Cells grown on glass coverslips were incubated with trastuzumab (10 μg/ml) or T-DM1 (1 μg/ml) at 37°C for 30 min. After washing, cells were fixed with 3.7% formaldehyde and permeabilized with 0.1% Triton-X 100. The fixed cells were incubated with rabbit anti-caveolin-1 antibody (1:100 dilution in PBS/0.1% Triton-100/3% BSA) at room temperature for 1 hr and then incubated with Cy3-conjugated anti-rabbit secondary antibodies (1:200 dilution in PBS/0.1% Triton-100/3% BSA) at room temperature for 1 hr. The presence of trastuzumab and T-DM1 was revealed by a Cy2-conjugated anti-human secondary antibody. Coverslips were mounted with Gel Mount aqueous mounting medium (Sigma, St. Louis, MO, USA). Images were acquired using a Zeiss LSM 510 META confocal microscope with a 63× objective (1.4 oil).

### Cell viability using a trypan blue exclusion assay

Cells were seeded into 24 well plates at a density of 0.1 x 10^6^ cells/ml and incubated alone or in 10 μg/ml trastuzumab (Herceptin, Roche Ltd., Basel, Swiss), 1 μg/ml T-DM1 (Kadcyla, Roche Ltd., Basel, Swiss), or 1μg/ml paclitaxel (Phyxol, Sinphar Ltd., I-Lan, Taiwan) for 72 hr [37]. At the end of this period, 0.1 ml of 0.4% trypan blue and deionized water (1:1) was added to the control and treated tubes to estimate the number of dead cells. Cell viability was estimated with a hemocytometer. Dead cells were stained blue, while live cells remained unstained. The cell mortality was expressed as the percentage of trypan blue-positive cells compared to the total number of cells. The viability percentage was presented by the number of live cells divided by the number of untreated control cells ×100.

### Cell apoptosis analysis

Cell apoptosis was analyzed at 48 hours after drug treatment [11]. Pro-survival protein Bcl-2 was determined by Western blot. A measurement of the extent of apoptosis was carried out using an annexin V detection kit (Invitrogen, ThermoFisher, Waltham, MA, USA) to label cell surface phosphatidylserine of apoptotic cells. Briefly, treated cells were trypsinized and washed twice with phosphate-buffered saline, then suspended in binding buffer (10 mM HEPES, pH 7.4, 140 mM NaCl and 2.5 mM CaCl_2_). To each 100 μl cell suspension was added 5 μl annexin V conjugated to fluorescein isothiocyanate (FITC), cells were incubated at room temperature for 30 min. Stained cells were analyzed immediately by flow cytometry. To further monitor the status of cell apoptosis, T-DM1 treated cells were live stained with annexin V and propodium iodide (PI) together, co-staining images were taken by inverted fluorescence microscope. Annexin V stained cells with PI positive staining were excluded as necrotic cells, apoptotic cells with only annexin V staining were counted and divided by total cells as the percentage of apoptosis. Pan-caspase inhibitor Z-VAD-FMK (Enzo, Farmingdale, NY, USA) with working concentration 20 μM was introduced together with T-DM1 for rescue experiment, cell viability was determined as described in cell viability assay.

### Statistics

Results are expressed as mean ± standard deviation. A Student t-test was used to compare continuous variables. Differences at the * *P* <0.05, ** *P* <0.01 and *** *P* <0.001 were considered statistically significant. Tukey Kramer test was used for multiple comparisons, different letters represent significant difference at the *P* <0.01 level. For cell culture experiments, at least three independent experiments were performed.

## Results

### Expression of caveolin-1 in breast cancer specimens

In this study, the caveolin-1 expression pattern was examined in 32 breast cancer patients. Imnunohistochemical staining showed that caveolin-1 was concentrated at the membrane, and a diffuse cytoplasmic staining pattern was observed also, in ductal epithelial cells with different expression levels (Fig 1). Twenty-two cases (68.75%) of invasive ductal carcinoma showed high levels (score≥1.5) of caveolin-1 staining, whereas low expression of caveolin-1 (score <1.5) was noted in 10 cases (31.25%) (S1 Table). The expression of caveolin-1 did not differ between HER-2 (+) and HER-2 (-) tissue (1.97 ± 0.70 vs 1.77 ± 0.77, *P* = 0.45). Similarly, the scores in the ER (+) and PR (+) groups did not differ to those of the ER(-) and PR(-) groups (1.80 ± 0.77 vs 1.93 ± 0.69, *P* = 0.63 and 1.77 ± 0.82 vs 1.91 ± 0.66, *P* = 0.59) (S2 Table). In addition, in the study of Western blot protein evaluation, 8 (72.72%) of the 11 HER-2 positive breast cancer patients were found to have a high expression of caveolin-1 in tumor tissue than in the non-tumor part of the patient (S3 Table). Sample of non-tumor site specimens all showed high expression of caveolin-1 with heterogeneous distribution in ductal epithelial.

### Endocytic protein expression in breast cancer cell lines

**Fig 2:**
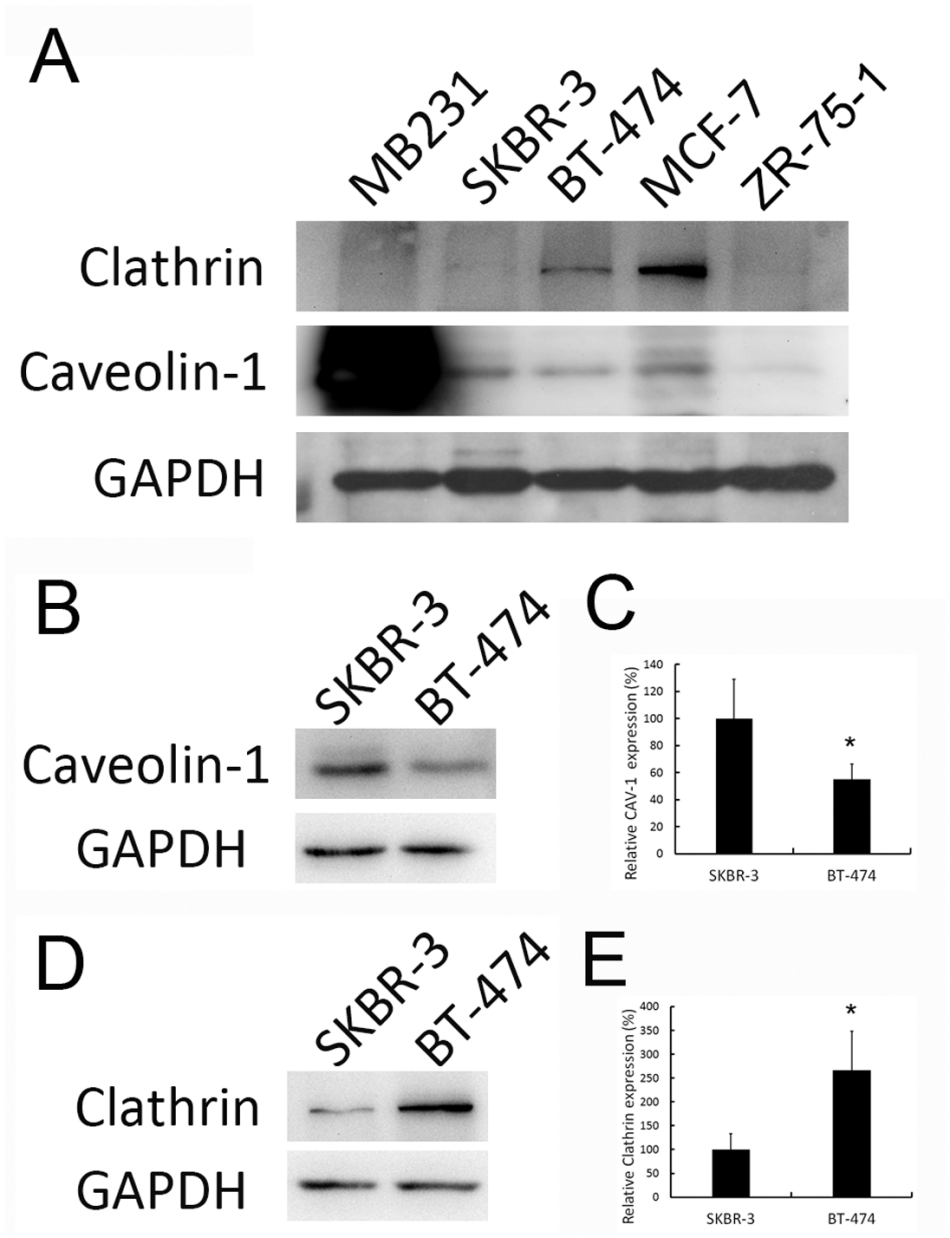
Caveolin-1 and clathrin expression in breast cancer cell lines. (A) Western blot analysis of cell lysates of breast cancer cell lines, ZR-75-1,MDA-MB-231, MCF-7, SKBR-3, and BT-474 with antibodies against caveolin-1 and clathrin. GAPDH was used as an internal control. Caveolin-1 expression level was highest in MDA-MB-231 cells, followed by MCF-7, SKBR-3, BT-474, and then ZR-75-1 cells. Detail caveolin-1 (B-C) and clathrin (D-E) expression in SKBR-3 and BT-474 cells were shown. Quantifications from at least three independent experiments were performed, value = mean ± SD, * *P* <0.05. CAV-1 indicates caveolin-1.

The expression of the two major endocytic proteins clathrin and caveolin-1 was examined in the breast cancer cell lines, MDA-MB-231, MCF-7, SKBR-3, BT-474, and ZR-75-1. Western blotting demonstrated that clathrin and caveolin-1 expression levels differed in these breast cancer cell lines: caveolin-1 was highly expressed in MDA-MB-231 cells. For HER-2 positive cells, caveolin-1 expression was higher in SKBR-3 than in BT-474 cells (Fig 2A, 2B and 2C, S1A Fig). Clathrin was more abundant in BT-474 cells than in SKBR-3 cells (Fig 2A, 2D and 2E, S1B Fig). Triple negative MDA-MB-231, HER-2 positive (3+) SKBR-3, and BT-474 cell lines were therefore chosen for the following experiments to monitor the effect of caveolin-1 on drug treatment.

### Internalization and colocalization of trastuzumab and T-DM1 with caveolin-1 in HER-2 positive breast cancer cells

**Fig 3:**
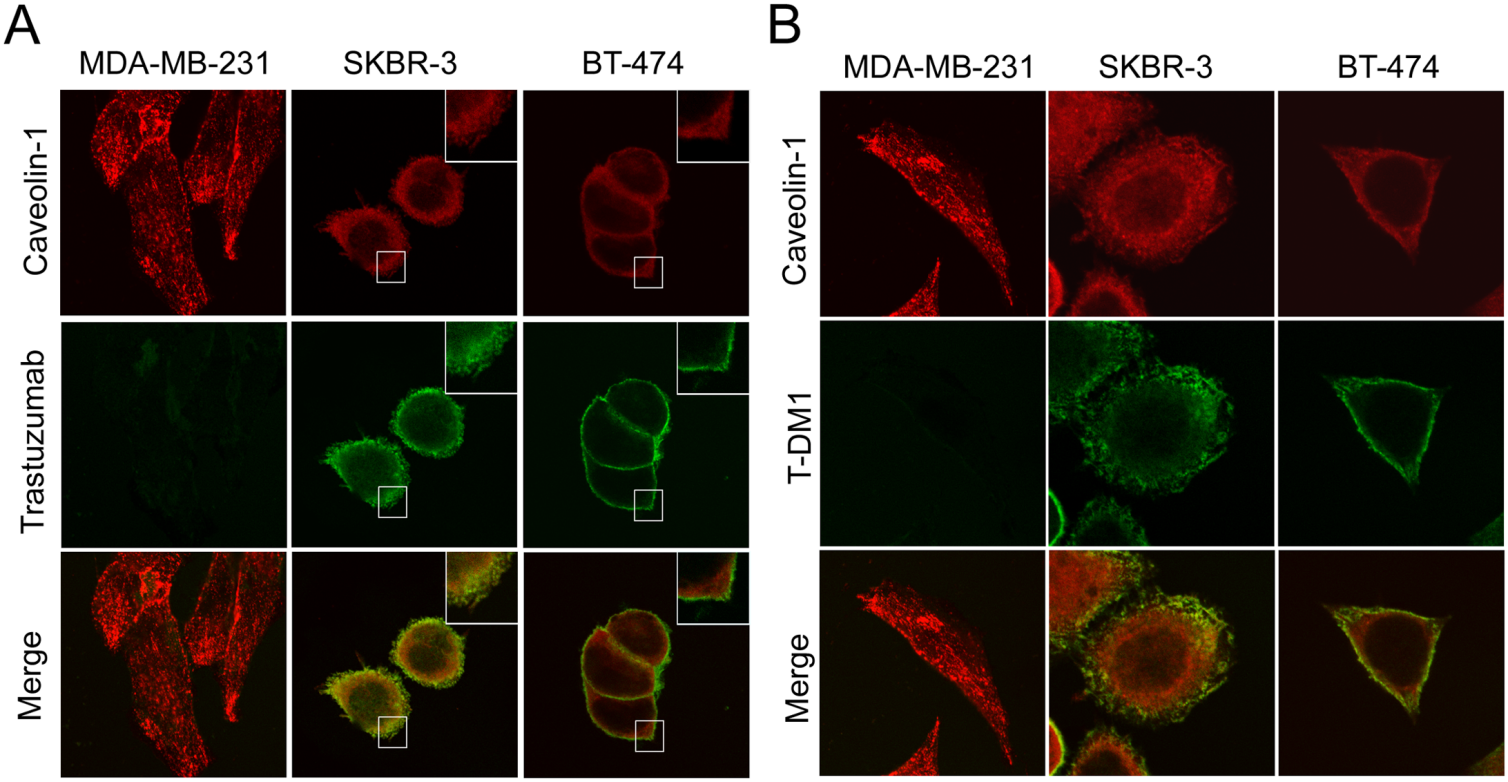
Caveolin-1 expression promoted trastuzumab and T-DM1 internalization into the cells. MDA-MB-231, SKBR-3, and BT-474 cells were incubated with trastuzumab (A) or T-DM1 (B) for 30 minutes. Cells were then fixed and the caveolin-1 was detected using an antibody against caveolin-1. The presence of caveolin-1 and trastuzumab/T-DM1 were then revealed by immuno-confocal microscopy. Green color indicates trastuzumab/T-DM1, red color indicates caveolin-1. HER-2 positive SKBR3 cells, which have a high caveolin-1 expression, induced trastuzumab and T-DM1 internalization into the cell and these were both colocalized with caveolin-1.

MDA-MB-231, SKBR-3 and BT-474 cells were treated with trastuzumab or T-DM1 for 30 min. Trastuzumab, T-DM1, and endogenous caveolin-1 were revealed by immunofluorescence microscopy. Double immune staining micrographs demonstrated that neither trastuzumab nor T-DM1 was present in MDA-MB-231 cells, which did display an apparent caveolin-1 staining pattern (Fig 3A and 3B, left lane). The intensity of caveolin-1 staining was higher in SKBR-3 than BT-474 cells, and showed a clear cytosolic distribution (Fig 3A and 3B, middle lane). Trastuzumab and T-DM1 were colocalized with caveolin-1 in the SKBR-3 cell membrane and cytosol; however, in BT-474 cells, most of the trastuzumab and T-DM1 was localized only in the cell membrane (Fig 3A and 3B, right lane). These results suggest that trastuzumab and T-DM1 were bound to HER-2 positive cells, and that the internalization of trastuzumab and T-DM1 was associated with caveolin-1 expression.

### Caveolin-1 expressing HER2 positive cells were sensitive to T-DM1 treatment

**Fig 4:**
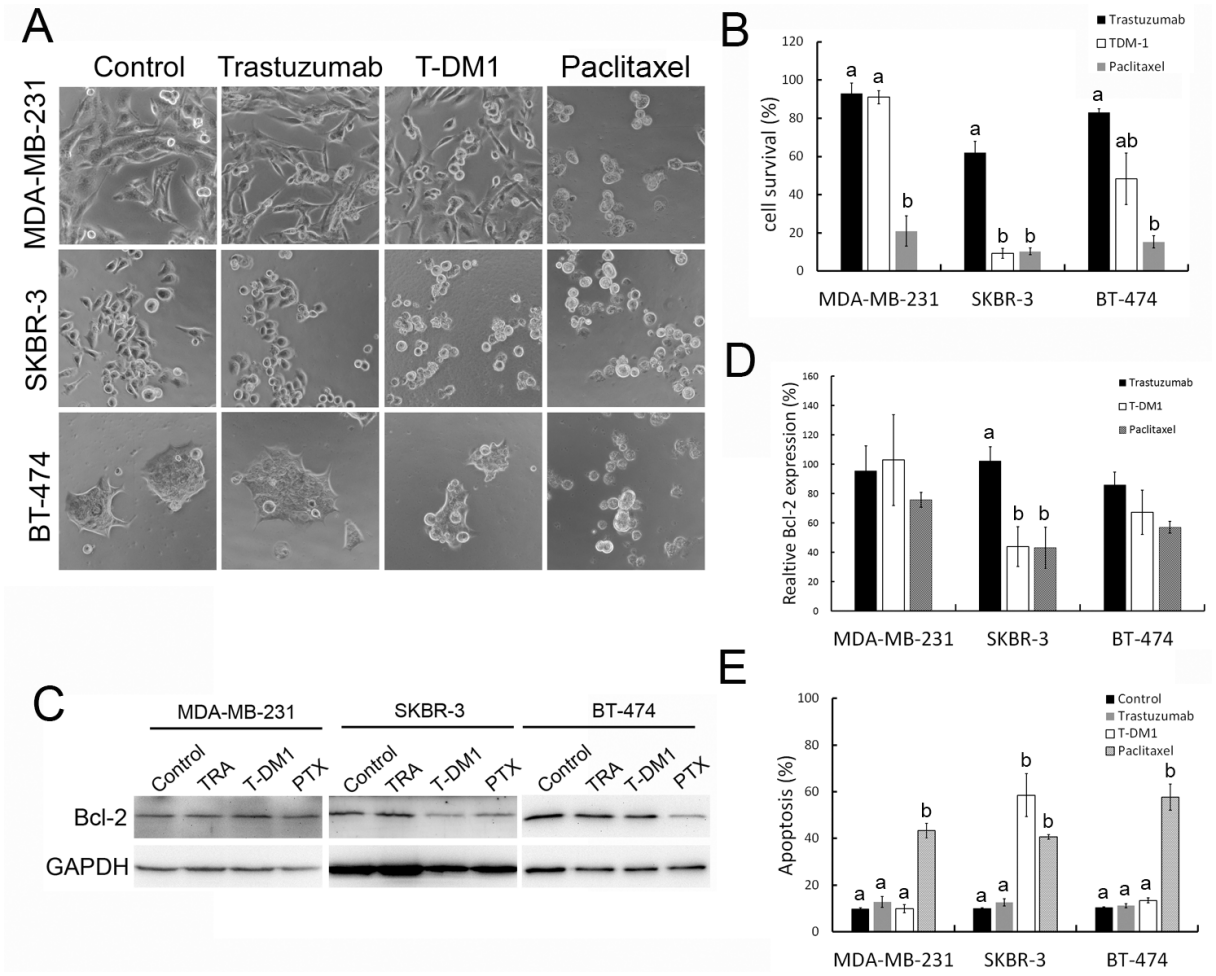
Caveolin-1 expressing HER-2 positive cells were sensitive to T-DM1 treatment. MDA-MB-231, SKBR-3, and BT-474 cells were treated with trastuzumab (10 μg/ml), T-DM1 (1 μg/ml) or paclitaxel (1 μg/ml) for 72 hours. (A) phase contrast images and (B) cell survival graph showing that paclitaxel causes cell toxicity in all cell types. SKBR-3 cells were most sensitive to T-DM1 treatment when compared to trastuzumab treatment. (C) Western blot analysis of the pro-survival marker Bcl-2, showing that T-DM1 treatment caused SKBR-3 cell damage following an apoptotic process. TRA indicates trastuzumab, PTX indicates paclitaxel. (D) Quantification of Bcl-2 expression levels from (C). (E) The quantification of cell apoptosis was determined by annexin V staining. Values in (B), (D) and (E) were mean ± SD. For each cell line, comparisons of drug treatments were achieved by Tukey Kramer method, statistic values with the different letter are significantly different (P <0.01). At least three independent experiments were performed for all quantifications.

Phase contrast micrographs of MDA-MB-231, SKBR-3, and BT-474 cells treated with trastuzumab, T-DM1, and paclitaxel demonstrated that paclitaxel treatment caused the greatest cell toxicity in each of the three cell types. Trastuzumab treatment also had a cytotoxic effect on HER-2 positive SKBR-3 and BT-474 cells; however, T-DM1 treatment caused more cytotoxicity in SKBR-3 cells than in BT-474 cells (Fig 4A). The results of the cell viability assay showed that SKBR-3 was significantly more sensitive to T-DM1 when compared to trastuzumab treatment (Fig 4B, *P* <0.01). The cytotoxic efficacy of T-DM1 was similar to paclitaxel treatment (Fig 4B).

It was demonstrated that T-DM1 induced cell toxicity through apoptosis at 48 hours [11, 32]. In this study, the pro-survival protein Bcl-2 which functions in negative regulation of many caspases activity during apoptosis was examined. In the result, lower level of Bcl-2 was detected in T-DM1 treated SKBR-3 cells via Western blot (Fig 4C and 4D). The percentage of apoptosis, represented by annexin V staining (Fig 4E), was significantly increased in SKBR-3 cells treated with T-DM1 (*P* <0.01), whereas, fewer necrotic cells were found with Propodium iodide (PI) staining (S2 Fig). Furthermore, drug sensitivity to T-DM1 was decreased when SKBR-3 was treated with pan-caspase inhibitor, Z-VAD-FMK (S3 Fig), this result is based on the 1μg/ml concentration of T-DM1 treatment, and however, the level of T-DM1 induced cell toxicity may be different dependent on T-DM1 concentration. These results indicate that HER-2 positive SKBR-3 cells, which have a higher caveolin-1 expression, are sensitive to T-DM1 treatment.

### Caveolin-1 expression is required for T-DM1 Internalization

**Fig 5:**
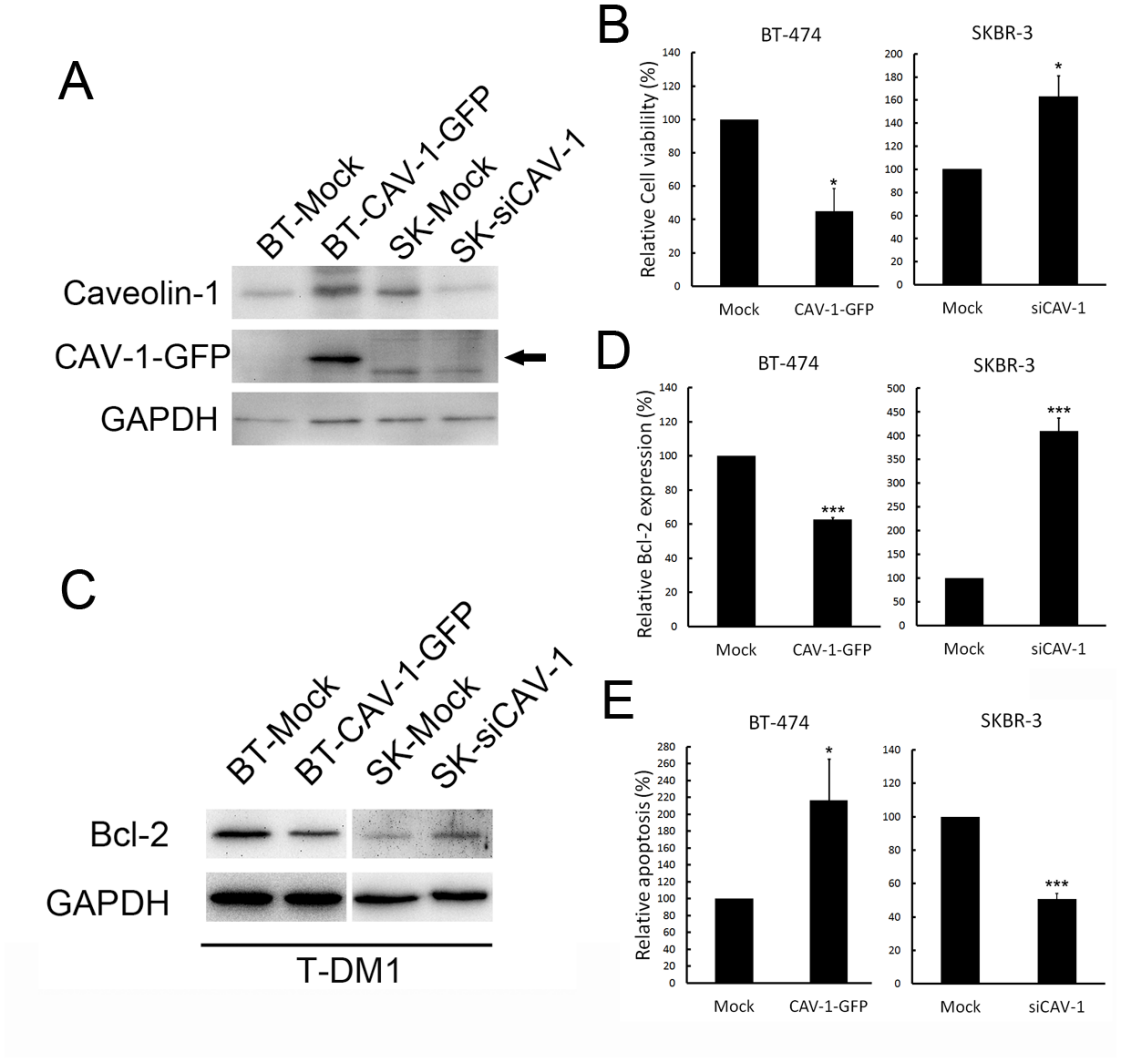
Caveolin-1 expression is required for T-DM1 Treatment. (A) Western blot analysis showing the overexpression and knockdown of caveolin-1 in BT-474 and SKBR-3 cells. BT indicated BT-474 cell; SK indicated SKBR-3 cell; si-CAV-1 indicated caveolin-1 siRNA. Arrow indicated overexpression of GFP tagged caveolin-1 (B) Cell survival, showing that when cells treated with T-DM1 (1 μg/ml) for 72 hours, the sensitivity of BT-474 cells to T-DM1 was increased when caveolin-1 was overexpressed, however, the SKBR-3 cells showed T-DM1 resistance when cellular caveolin-1 expression was inhibited by siRNA. (C-D) Western blot analysis of pro-survival marker Bcl-2, showing its inverse correlation with cell apoptosis (E). Quantifications were drawn from at least three independent experiments. Value = mean ± SD, * *P* <0.05, *** *P* <0.001.

To determine whether caveolin-1 plays role in T-DM1 internalization and influences drug sensitivity, a molecular strategy was applied to regulate caveolin-1 expression in breast cancer cells. BT-474 and SKBR-3 cells, which express relatively lower and higher caveolin-1 levels, respectively, were transfected with GFP-tagged caveolin-1 for caveolin-1 overexpression, or caveolin-1 siRNA for caveolin-1 knockdown, respectively. Western blotting demonstrated that caveolin-1 was overexpressed in GFP-caveolin-1 transfected BT-474 cells, and was suppressed in siRNA-treated SKBR-3 cells (Fig 5A, S4 Fig). The overexpression of caveolin-1 in BT-474 increased T-DM1 cytotoxicity, however, caveolin-1 knockdown in SKBR-3 cells significantly reduced T-DM1 efficacy (*P* <0.05) (Fig 5B). This T-DM1-induced cytotoxicity was associated with the level of Bcl-2 expression (Fig 5C and 5D), as shown by the ratio of apoptosis (Fig 5E). The cytotoxic effect of T-DM1 was negatively associated with Bc1-2 levels in both SKBR-3 and BT-474 cells, which express different levels of caveolin-1. The percentage of apoptosis measured by flow cytometry was associated with the chemosensitivity of the cells to T-DM1 induced by the overexpression of caveolin-1. Taken together, these data suggest that caveolin-1 is required for T-DM1 internalization and that this internalized conjugate further induces cell apoptosis.

## Discussion

Caveolin-1 regulates the formation of caveolae; the invaginated membrane structure with multiple transport and sorting functions in membrane endocytic system [38]. Caveolin-1 has been demonstrated to have an oncogenic or tumor suppressor role in breast cancer [29, 39]. It has also been suggested that the high expression of caveolin-1 in stromal cells has a protective effect against tumor progression in breast cancer and could be a good prognostic indicator [35, 40]. In our study, a high level of caveolin-1 expression was noted in 68.8% of breast cancer specimens. This incidence is similar to that of a study by Park et al with 130 breast cancer patients [35]. However, their caveolin-1 expression was correlated inversely with HER-2 expression status. This phenomenon was not observed in our series; possibly due to small sample size. Meta-analysis from 19 eligible studies included a total of 5,926 patients with a median number of 312 patients per study failed to recognize that caveolin-1 expression in tumor cells as a predictive marker for breast cancer prognosis. [41].

However, caveolin-1 may participate in different signaling pathways depending on cell type and the genetic background of the breast cancer [42, 43]. In our study, caveolin-1 expression varied in different breast cancer cell lines: triple negative MDA-MB-231 cells showed the greatest caveolin-1 expression, while HER-2 positive SKBR-3 and BT-474 cells showed relatively moderate and lower caveolin-1 expressions, respectively. This observation agreed with the results of a previous study in which the levels of caveolin-1 mRNA level were shown to be associated with DNA methylation [44]. Furthermore, our results demonstrated that the expression of caveolin-1 can improve T-DM1 internalization and drug efficacy, although tumor stage and the expression of molecular effectors may affect the role of caveolin-1 during tumor progression [45]. The efficacies of trastuzumab and T-DM1 in breast cancer cell lines have been demonstrated previously [11, 37]. According to Barok's protocol [37], our data also showed trastuzumab treatment had a similar efficacy on 72 hours treatment in SKBR-3 and BT-474 cell lines, based on HER-2 expression, while SKBR-3 cells, which have a higher caveolin-1 expression, were more sensitive to T-DM1. The sensitivity of T-DM1 was associated with caveolin-1 expression. Caveolin-1 has been shown to improve trastuzumab internalization [31]. In this study, the role of caveolin-1 in T-DM1 internalization and its dramatic cytotoxicity was demonstrated for the first time.

Molecular mechanisms have been suggested for the antitumor activity of T-DM1, including disruption of the microtubule network of target cells, the induction of apoptosis, mitotic catastrophe, and defective intracellular trafficking of HER-2 [32, 37]. Our results revealed effective cytotoxicity of paclitaxel in the treatment of different breast cancer cells which were correlated with cell apoptosis. Although the Tukey statistic showed no significant difference of Bcl-2 expression in MDA-MB-231 and BT-474 cells when trastuzumab, T-DM1 and paclitaxel treatment were compared. However, paclitaxel treatment showed significant reduction of Bcl-2 expression in MDA-MB-231 (*P* <0.01) and BT-474 cells (*P* <0.01) when compared to trastuzumab treatment by *t*-test. Since the chemotherapeutic drugs of taxane group have high incidence of side effect in clinical application [46], our study focused on the mechanism of caveolin-1 induced chemosensitivity of T-DM1 that also associated with a reduction in Bcl-2 expression and an increase in the percentage of apoptosis. The expression of Bcl-2 protein was demonstrated to be associated with its transcription level [47].

Endocytosis has been recognized as the most significant pathway in the downregulation of EGFR by removing the receptor from cell surface for degradation in lysosomes [48]. Recently, the dominant opinion is that trastuzumab causes the endocytic downregulation of HER-2 [49–51]. Although the endocytic downregulation of EGFR has been largely attributed to clathrin-dependent endocytosis [52], other endocytic pathways have also been proposed in recent years, especially following stimulation with high concentration of EGF. In human mammary glands, the EGF concentration is greater than 100 ng/ml [53]. In a study by Sigismud et al. [54], the EGFR was found to become ubiquitinated and was internalized by caveolae at high concentration of EGF (20 ng/ml). The dynamics of membrane receptors are regulated by vesicle trafficking systems [18], therefore, the trafficking route of receptor-bound antibodies affects the antibody half-life in the cell and determines the drug efficacy.

The action of trastuzumab is dependent on HER-2 signaling through Src hyperactivation, which further inhibits PTEN and its downstream PI3K/AKT signaling in cell proliferation. Patients with a lack of PTEN, or compensatory HER-3 or IGF1-R activation, may be resistant to trastuzumab treatment [55]. Our results, indeed, were consistent with the theory that the efficacy of T-DM1 is mainly through the action of DM1 derivative that was released by the degradation of T-DM1, but that HER-2 expression is also required for T-DM1 treatment [11]. In addition, we confirmed caveolin-1 overexpression could be used to enhance T-DM1 uptake. However, a follow-up study on a second independent dataset is required to validate the correlation between the expression of caveolion-1, as well as drug sensitivity, with the response to T-DM1 clinically.

## Conclusion

This study demonstrated the role of endocytic protein caveolin-1 in T-DM1 internalization and improved cytotoxicity in breast cancer cells. The expression of caveolin-1 in HER-2 positive breast cancer cells could be a potential biomarker to predict the efficacy.

## Supporting Information

S1 Table Expression of HER-2, ER, PR and caveolin-1 in 32 breast cancer patients. (DOCX)

S2 Table The association of caveolin-1 expression with HER-2, ER or PR in patients by IHC. (DOCX)

S3 Table The association of caveolin-1 expression with HER-2, ER or PR in patients by Western blot. (DOCX)

S1 Fig Quantification of caveolin-1 and clathrin expression in different breast cancer cells. Western blot analysis of cell lysates of breast cancer cell lines, ZR-75-1,MDA-MB-231, MCF-7, SKBR-3, and BT-474 with antibodies against caveolin-1 (A) and clathrin (B). GAPDH was used as an internal control. Value = mean ± SD from at least three independent experiments.
(TIF)

S2 Fig T-DM1 induced cell apoptosis in SKBR-3 cells. MDA-MB-231, SKBR-3, and BT-474 cells were treated with T-DM1 (1 μg/ml) for 48 hours. (A) Cells were live stained with annexin V and propodium iodide (PI), double stained images were taken by inverted fluorescence microscope. Green color indicates annexin V positive apoptotic cell, red color indicates PI positive necrotic cell (arrows). (B) Quantification of annexin V positive/ PI negative apoptosis from images. Value = mean ± SD from three independent experiments. **p<0.01.
(TIF)

S3 Fig T-DM1 drug efficacy was suppressed by pan-caspase inhibitor in SKBR-3 cells. MDA-MB-231, SKBR-3 and BT-474 cells were treated with 1μg/ml T-DM1 together with 20μM pan-caspase inhibitor Z-VAD-FMK for 72 hours. Cell viability was determined and showed drug sensitivity to T-DM1 in SKBR-3 cells was suppressed. Value = mean ± SD from three independent experiments. *p<0.05.
(TIF)

S4 Fig Quantification of caveolin-1 knockdown efficiency in SKBR-3 cells. SKBR-3 cells transfected with caveolin-1 siRNA for 48 hours were than subjected for Western blot with caveolin-1 antibody, GAPDH was used as an internal control. Caveolin-1 expression from Western blot result was quantified. GAPDH was used as an internal control. Value = mean ± SD from at least three independent experiments. *p<0.05.
(TIF)
