# Supplementary material for: Caveolin-1 Dependent Endocytosis Enhances the Chemosensitivity of HER-2 Positive Breast Cancer Cells to Trastuzumab Emtansine (T-DM1)
Source: PLoS One. 2015 Jul 14;10(7):e0133072. doi: 10.1371/journal.pone.0133072 (PMC4501549; doi:10.1371/journal.pone.0133072)
Supplement: S1 Table — (DOCX) [file pone.0133072.s001.docx]

S1 Table. Expression of HER-2, ER (estrogen receptor), PR (progesterone receptor) and Cav-1 (caveolin-1) in 32 breast cancer patients

| Patient | Age | Stage | ER | PR | HER-2 | Grade | CAV-1  Tumor | CAV-1  Stroma |
| --- | --- | --- | --- | --- | --- | --- | --- | --- |
| 1 | 55 | 2 | － | － | － | 3 | 0.5 | 1.0 |
| 2 | 44 | 2 | － | － | － | 3 | 1.4 | 1.4 |
| 3 | 42 | 3 | ＋ | － | － | 3 | 1.8 | 1.7 |
| 4 | 49 | 2 | ＋ | ＋ | ＋ | 2 | 2.7 | 2.3 |
| 5 | 70 | 3 | ＋ | ＋ | － | 2 | 0.3 | 0.8 |
| 6 | 42 | 3 | － | － | ＋ | 3 | 1.6 | 1.8 |
| 7 | 35 | 2 | ＋ | ＋ | ＋ | 2 | 0.3 | 1.1 |
| 8 | 47 | 3 | ＋ | ＋ | ＋ | 2 | 1.6 | 1.4 |
| 9 | 44 | 2 | ＋ | ＋ | － | 2 | 1.9 | 1.5 |
| 10 | 47 | 3 | ＋ | ＋ | － | 2 | 1.6 | 1.4 |
| 11 | 41 | 2 | ＋ | ＋ | － | 2 | 2.6 | 1.4 |
| 12 | 55 | 1 | ＋ | ＋ | － | 1 | 1.4 | 1.4 |
| 13 | 85 | 3 | － | － | ＋ | 3 | 1.8 | 1.6 |
| 14 | 68 | 2 | ＋ | － | － | 2 | 2.7 | 1.8 |
| 15 | 50 | 3 | ＋ | ＋ | － | 2 | 2.4 | 2.0 |
| 16 | 39 | 3 | ＋ | ＋ | － | 2 | 1.8 | 1.6 |
| 17 | 48 | 2 | ＋ | ＋ | ＋ | 2 | 2.8 | 1.6 |
| 18 | 55 | 2 | － | － | ＋ | 2 | 2.4 | 2.7 |
| 19 | 61 | 2 | ＋ | ＋ | － | 2 | 1.4 | 1.4 |
| 20 | 40 | 3 | ＋ | ＋ | － | 3 | 1.9 | 1.4 |
| 21 | 55 | 3 | － | ＋ | － | 3 | 2.9 | 2.1 |
| 22 | 47 | 1 | ＋ | ＋ | － | 1 | 2.4 | 2.2 |
| 23 | 48 | 2 | ＋ | ＋ | － | 2 | 0.7 | 1.9 |
| 24 | 44 | 3 | ＋ | ＋ | － | 1 | 1.4 | 1.8 |
| 25 | 72 | 3 | ＋ | － | － | 3 | 2.9 | 1.7 |
| 26 | 70 | 3 | － | － | ＋ | 3 | 2 | 1.8 |
| 27 | 33 | 2 | － | － | ＋ | 3 | 2.4 | 1.7 |
| 28 | 59 | 2 | － | － | ＋ | 3 | 1.7 | 1.0 |
| 29 | 45 | 3 | ＋ | － | － | 3 | 1.1 | 1.5 |
| 30 | 63 | 1 | ＋ | － | － | 3 | 1.4 | 1.2 |
| 31 | 63 | 3 | － | － | － | 3 | 2.6 | 1.2 |
| 32 | 56 | 3 | ＋ | － | ＋ | 3 | 2.4 | 1.7 |

|  | HER-2 (+) | ER(+) | PR(+) | Cav-1(+)  tumor | Cav-1(+)  stroma |
| --- | --- | --- | --- | --- | --- |
| Stage 1 | 0/3 (0%) | 3/3 (100%) | 2/3 (66.67%) | 1/3 (33.33%) | 1/3 (33.33%) |
| Stage 2 | 6/13 (46.15%) | 8/13 (61.54%) | 7/13 (53.85%) | 8/13 (61.54%) | 7/13 (53.85%) |
| Stage 3 | 5/16 (31.25%) | 11/16 (68.75%) | 8/16 (50%) | 13/16 (81.25%) | 11/16 (68.75%) |
| Total patients | 11/32 (34.38%) | 22/32 (68.75%) | 17/32 (53.13%) | 22/32 (68.75%) | 19/32 (59.38%) |

The symbol (+) in HER-2, ER and PR indicate the positive expression according to the clinical reports from pathology department in Cheng-Ching hospital. The “I index” of caveolin-1 greater than median value 1.5 was considered as positive.
