# Supplementary material for: Caveolin-1 Dependent Endocytosis Enhances the Chemosensitivity of HER-2 Positive Breast Cancer Cells to Trastuzumab Emtansine (T-DM1)
Source: PLoS One. 2015 Jul 14;10(7):e0133072. doi: 10.1371/journal.pone.0133072 (PMC4501549; doi:10.1371/journal.pone.0133072)
Supplement: S2 Table — (DOCX) [file pone.0133072.s002.docx]

S2 Table. The association of caveolin-1 expression with HER-2, ER or PR in breast cancer patients by immunohistochemistry

|  | Cav-1 expression (I index) | |
| --- | --- | --- |
| HER-2 (+) | 1.97±0.7 | *p*=0.45 |
| HER-2 (-) | 1.77±0.77 |  |
| ER (+) | 1.80±0.77 | *p*=0.63 |
| ER (-) | 1.93±0.69 |  |
| PR (+) | 1.77±0.82 | *p*=0.59 |
| PR (-) | 1.91±0.66 |  |

I index of caveolin-1 expression = value ±SD
