# Supplementary material for: Caveolin-1 Dependent Endocytosis Enhances the Chemosensitivity of HER-2 Positive Breast Cancer Cells to Trastuzumab Emtansine (T-DM1)
Source: PLoS One. 2015 Jul 14;10(7):e0133072. doi: 10.1371/journal.pone.0133072 (PMC4501549; doi:10.1371/journal.pone.0133072)
Supplement: S3 Table — (DOCX) [file pone.0133072.s003.docx]

S3 Table. The result of caveolin-1 expression by Western blot and the association with HER-2, ER or PR in breast cancer patients

|  | Cav-1 expression,  tumor>normal |
| --- | --- |
| HER-2 (+) | 8/11 (72.7%) |
| HER-2(-) | 17/21 (81%) |
| ER (+) | 18/22 (81.9%) |
| ER (-) | 7/10 (70%) |
| PR (+) | 14/17 (82.4%) |
| PR (-) | 11/15 (73.3%) |

Values indicate patients with higher caveolin-1 expression in tumor tissue than in the non-tumor part.
