# Supplementary figures and images for: Caveolin-1 Dependent Endocytosis Enhances the Chemosensitivity of HER-2 Positive Breast Cancer Cells to Trastuzumab Emtansine (T-DM1)
Source: PLoS One. 2015 Jul 14;10(7):e0133072. doi: 10.1371/journal.pone.0133072 (PMC4501549; doi:10.1371/journal.pone.0133072)

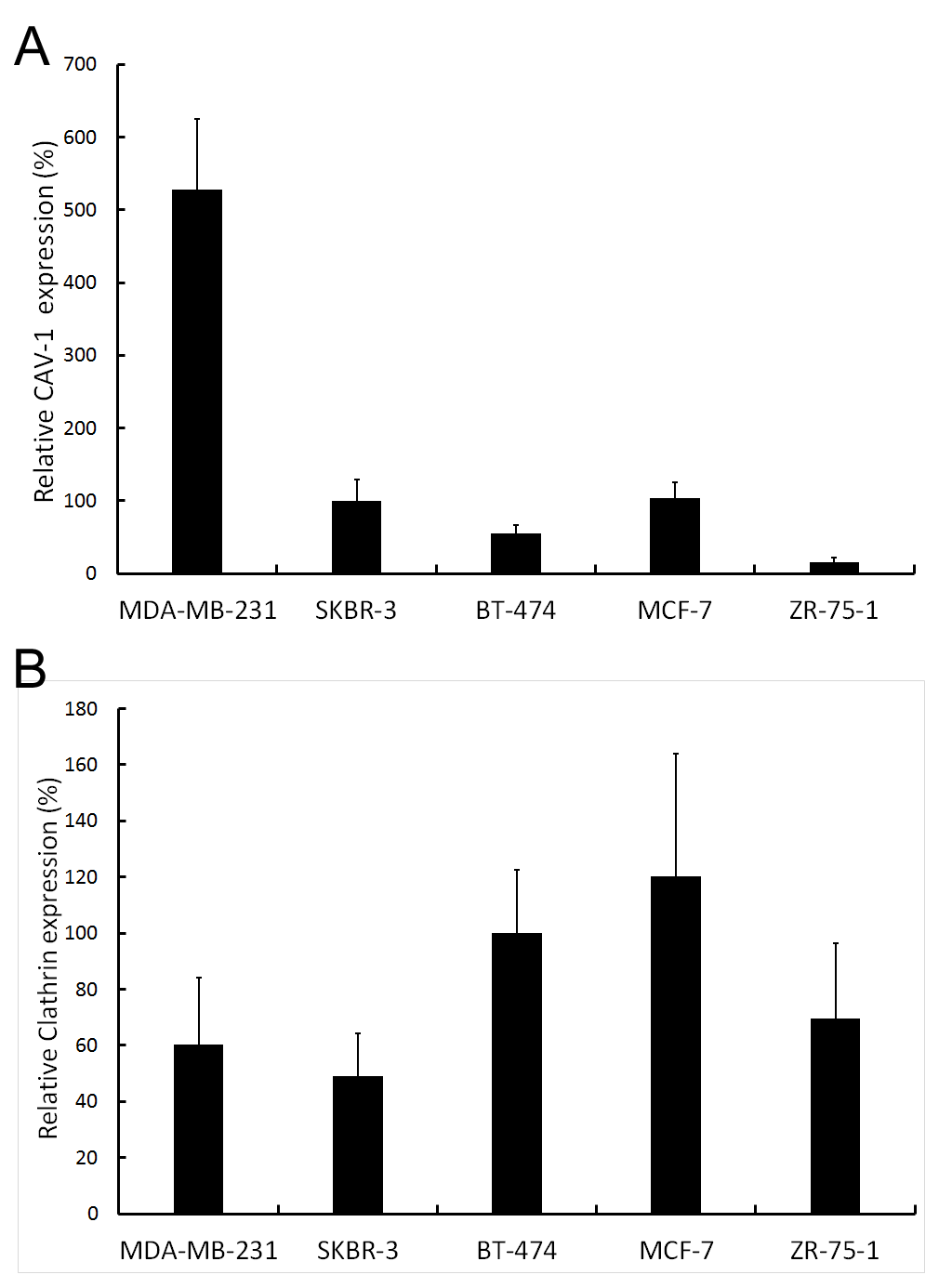

Supplement: S1 Fig — Western blot analysis of cell lysates of breast cancer cell lines, ZR-75-1,MDA-MB-231, MCF-7, SKBR-3, and BT-474 with antibodies against caveolin-1 (A) and clathrin (B). GAPDH was used as an internal control. Value = mean ± SD from at least three independent experiments. (TIF) [file pone.0133072.s004.tif]

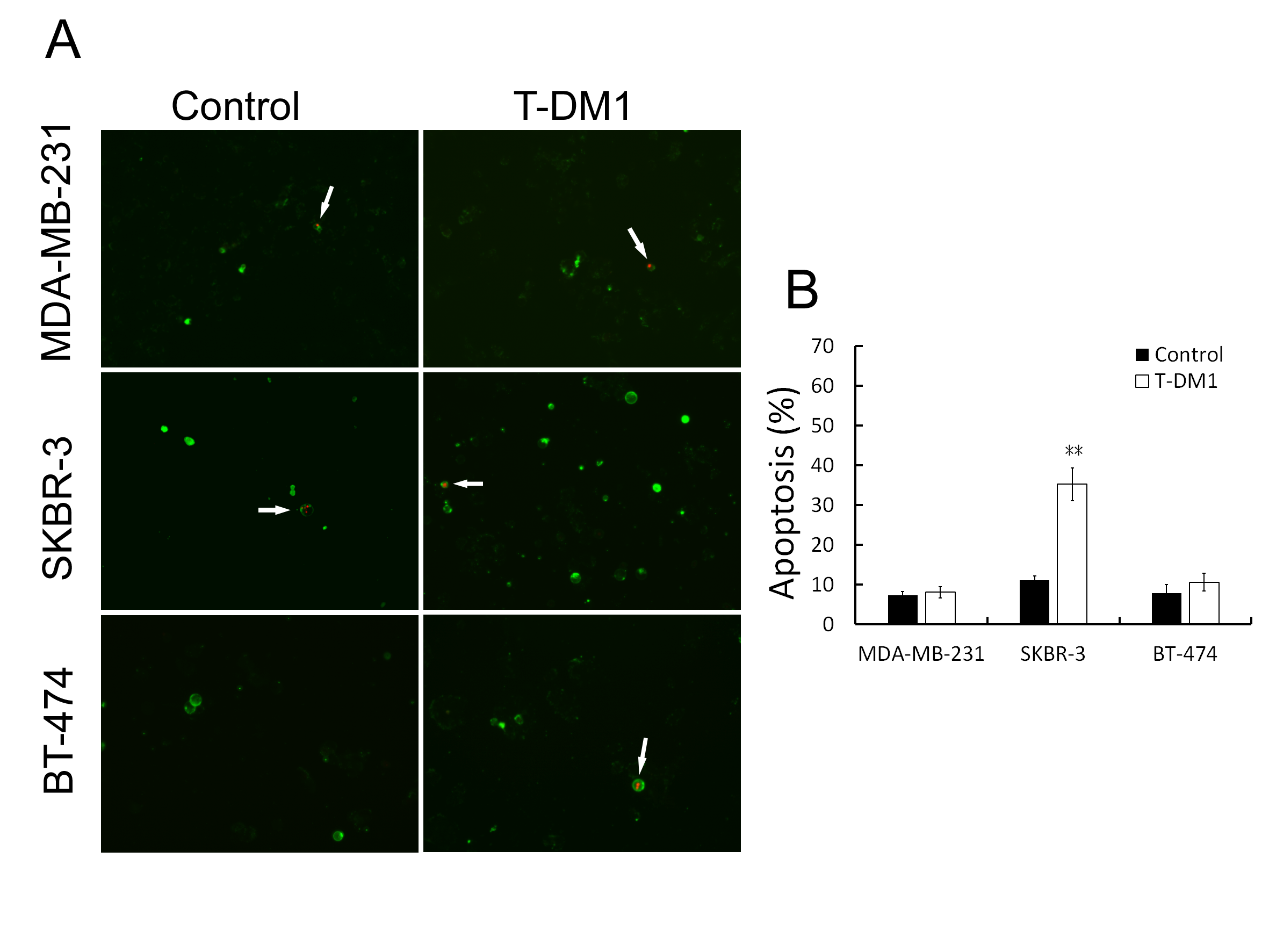

Supplement: S2 Fig — MDA-MB-231, SKBR-3, and BT-474 cells were treated with T-DM1 (1 μg/ml) for 48 hours. (A) Cells were live stained with annexin V and propodium iodide (PI), double stained images were taken by inverted fluorescence microscope. Green color indicates annexin V positive apoptotic cell, red color indicates PI positive necrotic cell (arrows). (B) Quantification of annexin V positive/ PI negative apoptosis from images. Value = mean ± SD from three independent experiments. **p<0.01. (TIF) [file pone.0133072.s005.tif]

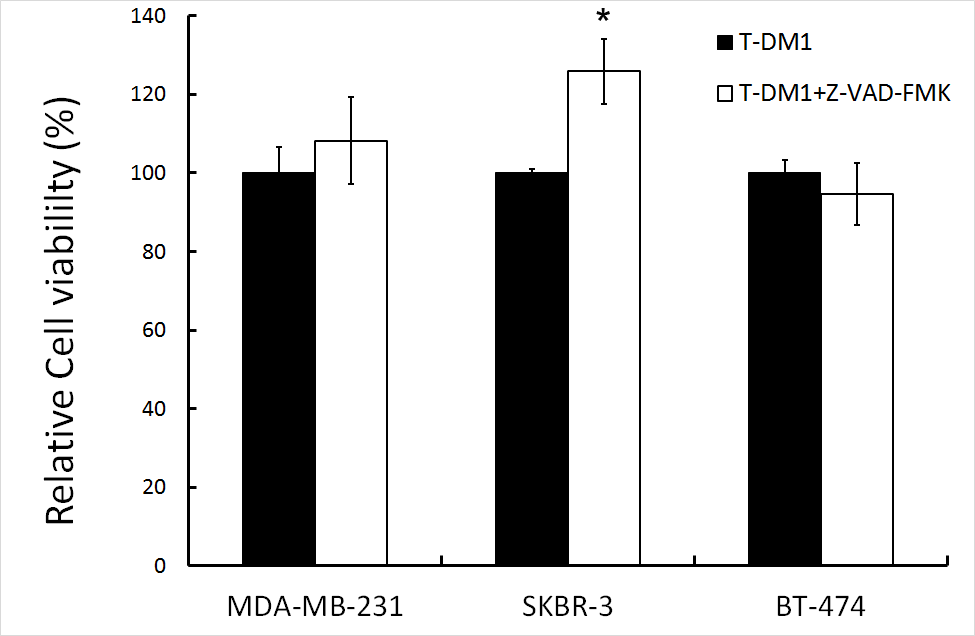

Supplement: S3 Fig — MDA-MB-231, SKBR-3 and BT-474 cells were treated with 1μg/ml T-DM1 together with 20μM pan-caspase inhibitor Z-VAD-FMK for 72 hours. Cell viability was determined and showed drug sensitivity to T-DM1 in SKBR-3 cells was suppressed. Value = mean ± SD from three independent experiments. *p<0.05. (TIF) [file pone.0133072.s006.tif]

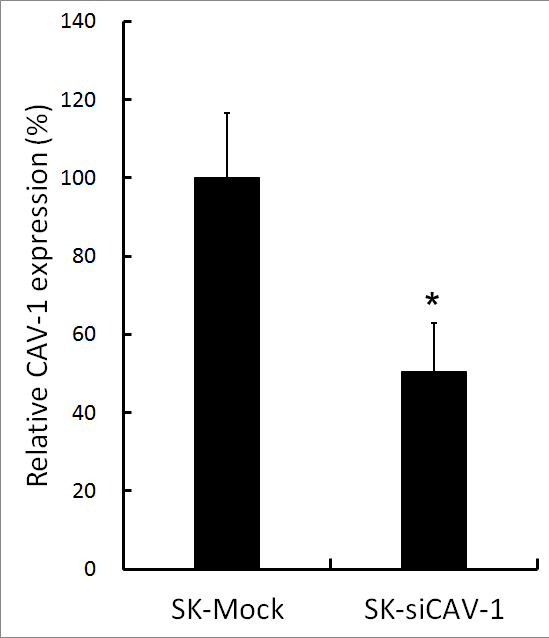

Supplement: S4 Fig — SKBR-3 cells transfected with caveolin-1 siRNA for 48 hours were than subjected for Western blot with caveolin-1 antibody, GAPDH was used as an internal control. Caveolin-1 expression from Western blot result was quantified. GAPDH was used as an internal control. Value = mean ± SD from at least three independent experiments. *p<0.05. (TIF) [file pone.0133072.s007.tif]
